# Synchronous presentation of acute acalculous cholecystitis and appendicitis: a case report

**DOI:** 10.1186/1752-1947-5-551

**Published:** 2011-11-14

**Authors:** Shaheel M Sahebally, John P Burke, Niamh Nolan, Amir Latif

**Affiliations:** 1 Department of General Surgery, St Columcille's Hospital, Loughlinstown, County Dublin, Ireland; 2 Department of Histopathology, St Columcille's Hospital, Loughlinstown, County Dublin, Ireland

## Abstract

**Introduction:**

Acute acalculous cholecystitis is traditionally associated with elderly or critically ill patients.

**Case presentation:**

We present the case of an otherwise healthy 23-year-old Caucasian man who presented with acute right-sided abdominal pain. An ultrasound examination revealed evidence of acute acalculous cholecystitis. A laparoscopy was undertaken and the dual pathologies of acute acalculous cholecystitis and acute appendicitis were discovered and a laparoscopic cholecystectomy and appendectomy were performed.

**Conclusion:**

Acute acalculous cholecystitis is a rare clinical entity in young, healthy patients and this report describes the unusual association of acute acalculous cholecystitis and appendicitis. A single stage combined laparoscopic appendectomy and cholecystectomy is an effective treatment modality.

## Introduction

Acute acalculous cholecystitis (AAC) is rare clinical entity traditionally associated with elderly patients with extensive co-morbidities or critically ill patients, such as those with burns or who have sustained trauma. We present a case of an otherwise healthy 23-year-old man who presented with acute right sided abdominal pain and had ultrasonographic evidence of both AAC and acute appendicitis.

## Case presentation

A 23-year-old unemployed Caucasian man presented to our Emergency Department with a twelve-hour history of severe right upper and lower quadrant pain. This pain originated in his epigastrium and was associated with nausea, multiple episodes of non-bilious vomiting and anorexia. His background history was unremarkable. He was on no regular medications, did not smoke and was a social drinker. On physical examination, he had a normal pulse and blood pressure but was pyrexic (38.5°C). An abdominal examination revealed tenderness in his right upper quadrant and right iliac fossa, guarding and rebound tenderness. Rovsing, obturator and psoas signs were negative. Laboratory investigations revealed an elevated white cell count of 14.3 × 10^9^/L, and slightly deranged liver function tests, namely a total bilirubin of 54 μmol/L and aspartate aminotransferase of 39 U/L with normal renal function and electrolytes. A dipstick of his urine showed 1+ bilirubin, 1+ blood and 4+ ketones. His Alvarado score was 10, consistent with appendicitis [1].

**Figure 1:**
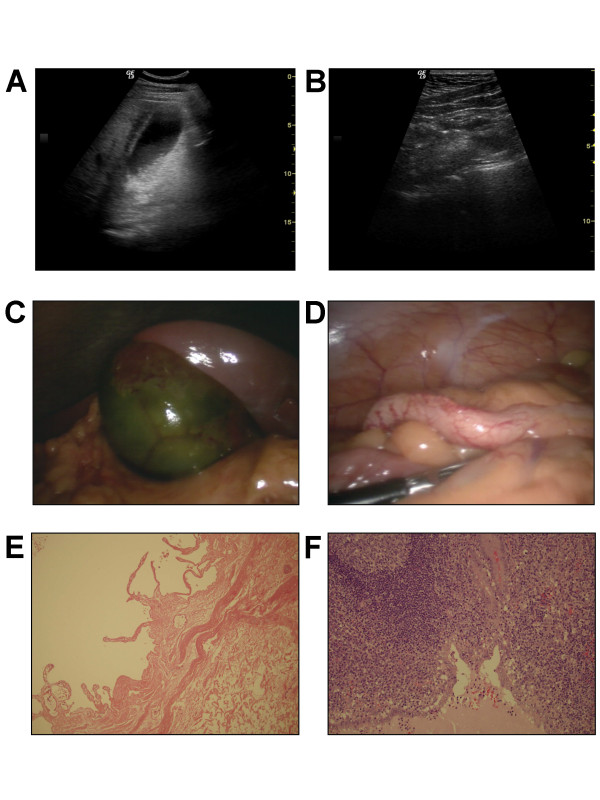
**Diagnostic, intra-operative and histological images**. Ultrasound images of **(A) **his gallbladder showing a thickened wall but no gallstones and **(B) **of his right iliac fossa, without evidence of free fluid and without visualization of his appendix. Intra-operative laparoscopy images demonstrating **(C) **a necrotic gallbladder and **(D) **acute appendicitis. Histological images demonstrating **(E) **gallbladder mucosa with acute inflammation with necrosis of the mucosa and submucosal thrombi and **(F) **acute appendicitis.

An ultrasound of his abdomen and pelvis revealed an inflamed, thick-walled gallbladder but no evidence of gallstones (Figure 1A). His appendix could not be visualised and there was no free fluid in the pelvis (Figure 1B). A diagnostic laparoscopy was performed, which revealed a gangrenous gallbladder with omental wrapping (Figure 1C) and an acutely inflamed appendix with thickened mesentery (Figure 1D). A combined laparoscopic cholecystectomy and appendectomy was performed. Histological examination of the resected gallbladder and appendix showed acute cholecystitis with diffuse inflammation of the gallbladder wall, edema and necrosis with extensive venous thrombi but no evidence of gallstones (Figure 1E) along with acute appendicitis (Figure 1F). Microbiological culture of the gallbladder bile revealed no bacterial growth. Our patient's postoperative course was unremarkable and he was discharged home two days later. At the latest follow-up, four months after surgery, he is well and without complaint.

## Discussion

AAC is a well-recognized but poorly understood clinical entity. Traditionally, it occurs in elderly patients with chronic debilitating disease or patients with critical illness, typically trauma or major burn injury. Whilst early case series associated AAC exclusively with critical illness [2], more recent reports demonstrate increasing *de novo *presentation of AAC in the absence of acute illness [3] and even in young, otherwise healthy patients without any predisposing factors [4]. The age of onset of AAC has been reported to be most commonly in the sixth decade [3]. The commonest postulated etiologies of AAC are bile stasis resulting in a change in bile composition, sepsis and ischemia [5]. In critically ill patients, AAC results from gallbladder ischemia, which may be secondary to shock due to hypovolemia or sepsis.

It has previously been noted that a hyperbilirubinemia occurs in acute appendicitis [6]. It has been proposed that appendicitis associated hyperbilirubinemia is due to bacterial translocation into the portal venous system, leading to altered bilirubin excretion. This, in combination with sepsis, may have precipitated AAC in our patient.

## Conclusion

AAC is a rare clinical entity in young, healthy patients and to the best of our knowledge, this represents the first report of AAC associated with acute appendicitis. A single stage combined laparoscopic appendectomy and cholecystectomy was an effective treatment modality in this case, although the timing of surgery for acute cholecystitis remains controversial, with some surgeons opting for interval cholecystectomy which carries a lesser risk of conversion to an open procedure or damage to the common bile duct, whereas other surgeons prefer early cholecystectomy to avoid failure of conservative management and to prevent disease recurrence. Surgical management of AAC in the end depends on the severity of the disease, physical status of the patient and the laparoscopic skill of the surgeon.

## Consent

Written informed consent was obtained from the patient for the publication of this case report and accompanying images. A copy of the written consent is available for review by the Editor-in-Chief of this journal.

## Competing interests

The authors declare that they have no competing interests.

## Authors' contributions

SS compiled and analyzed the patient's data and wrote the initial draft. JPB corrected the draft and assisted in the patient's operation. NN performed the histological examination of the resected gallbladder and appendix and contributed to writing the manuscript. AL performed the operation and reviewed the final manuscript. All authors read and approved the final manuscript.
